# FEM Analysis Validation of Rubber Hardness Impact on Mechanical and Softness Properties of Embossed Industrial Base Tissue Papers

**DOI:** 10.3390/polym14122485

**Published:** 2022-06-18

**Authors:** Joana Costa Vieira, António de O. Mendes, Marcelo Leite Ribeiro, André Costa Vieira, Ana Margarida Carta, Paulo Torrão Fiadeiro, Ana Paula Costa

**Affiliations:** 1 Fiber Materials and Environmental Technologies Research Unit (FibEnTech-UBI), University da Beira Interior, R. Marquês D’Ávila e Bolama, 6201-001 Covilhã, Portugal; ant.mendes@ubi.pt (A.d.O.M.); malribei@usp.br (M.L.R.); fiadeiro@ubi.pt (P.T.F.); anacosta@ubi.pt (A.P.C.); 2 Aeronautical Engineering Department, São Carlos School of Engineering, University of São Paulo, São Carlos 05508-060, SP, Brazil; 3 Center for Mechanical and Aerospace Science and Technologies (C-MAST-UBI), University da Beira Interior, R. Marquês D’Ávila e Bolama, 6201-001 Covilhã, Portugal; andre.costa.vieira@ubi.pt; 4 Forest and Paper Research Institute (RAIZ), R. José Estevão, Eixo, 3800-783 Aveiro, Portugal; ana.carta@thenavigatorcompany.com

**Keywords:** embossing prototype, FEM simulation, mechanical properties, optical visual inspection, rubber hardness, softness characterization, tissue paper

## Abstract

The embossing operation is one of the processes of tissue paper converting. The embossing parameters influence the final properties of tissue products, such as mechanical, softness, and bulk. In this study, the influence of the rubber hardness used against the embossing steel rolls with a pattern created by intaglio engraving was studied. Three different configurations of rubber plates stacking, each plate with different hardness, were studied. After embossing, mechanical properties, softness, and bulk were evaluated to analyze the effect of rubbers hardness on these properties. Furthermore, a Finite Element Model of the embossing operation was used that considered the same rubber plates stacking configurations used in experiments, and it was able to replicate the experimental results. This work led us to conclude that the configuration where two rubber plates with different hardness, where the rubber plate with higher hardness is in contact with the tissue paper sheet, has shown to be the best solution to obtain higher softness. These findings support the use of embossing operations rubber rolls with a low hardness internal layer and a high hardness external layer in industry. Thus, finite element models were also shown to be reliable tools to virtually test other configurations, such as, for example, three or more rubber plates with different hardness. Since embossing is one of the tissue paper transformation operations with the greatest impact on the key properties of the final product, this study allows the producer to optimize them by varying the hardness of the rubber roll, as well as its configuration.

## 1. Introduction

The embossing process comprises the step of passing the base tissue paper sheet through a nip formed by two rolls. A first steel roll, where the pattern is engraved, and a second steel roll with a rubber cover [1,2]. This operation is very important for tissue paper conversion, since causes an increase in volume, absorption capacity, and visual appeal, which are fundamental characteristics for the final consumer.

Tissue paper producers have, over the years, based the embossing and lamination process on two different types: nested (or point-to-valley) and point-to-point (PTP). The latter was first developed by Procter & Gamble in the 1960s, where great care is needed with the synchronization of the rollers to ensure correct point-to-point alignment [3]. Nystrand in 1970, instead, designed a method in which a point is aligned with a valley, where, although the rollers have yet to be synchronized to maintain the point-to-valley, this synchronization does not have to be perfect, because the alignment between a point and a valley allows a higher margin of error [3].

Over the years, many different solutions have emerged for the embossing and lamination process, in the end using one rubber cover steel roll. Today, this solution is the standard design for all converting machines. The hardness of the rubber cover has been increased to a value of 70 Sh-A. The current trend is to change the conventional rubber cover and introduce a double layer cover: a soft inner layer (with low hardness) and a hard outer layer (with high hardness), in order to obtain a greater degree of flexibility for the hard outer surface of the roll cover [3].

From the information provided by the manufacturers of embossing rollers with rubber cover, depending on the type of rubber and its hardness, some advantages in the maintenance and lifetime of this cover can be achieved (Table 1).

To select a rubber cover for an embossing roll, the user cannot worry only about which material works best. If the operating and environmental conditions do not affect the performance of the roll cover, then the decision focuses only on economic reasons. In most situations, the process and environmental conditions limit the type of material that can be considered in the roll covers [6]. Each rubber has its own characteristics, as shown in Table 1. Some are great for temperature resistance, others for chemical resistance, others for abrasion resistance, and others have a good lifetime. Therefore, one type of rubber may not be suitable for all applications. For example, polyurethane covers are great for abrasion resistance (long lifetime). As they are casted, they do not leave joint marks in the roll manufacturing process. These characteristics make the choice of polyurethane very desirable. However, this material has a relatively low maximum operating temperature (starting to soften at 71 °C and becoming liquid at 81 °C) [6].

To determine the hardness of the roll cover, it must be static, and the measure is carried out with the assistance of a durometer. This hardness is never determined at the nip of the embossing process and is always carried out before the roll with the cover is put into service. With the embossing process, the stresses that the rubber suffers, both in terms of pressure and temperature increase, would change the hardness value if it would be measured during the process. Thus, the determination of hardness using a durometer, by itself, is not a good indicator of the rubber’s performance under the conditions of embossing operation. The modulus of elasticity of the rubber is an alternative method to evaluate the performance of the rubber during the process, as it gives us information about the stiffness or flexibility of the material when exposed to forces during the process. Each rubber has a characteristic modulus value that is directly related to its molecular structure [6]. High elastic modules indicate materials with a more crystalline structure and, consequently, more stiff materials. On the other hand, lower elastic modules, indicate materials with a more amorphous structure and, consequently, more flexible materials [6,7]. In brief, two different rubbers can have the same hardness value, but have a completely different nip compression behavior, due to value of elastic modulus [6]. It is important to underline that hardness is directly associated with strength and not stiffness [7]. This performance of the embossing roll cover will influence the properties of embossed tissue paper and directly the final product.

It has long been recognized that the most important physical characteristics of tissue paper products are their strength, thickness, softness, and absorption [1,8,9,10]. Research and development efforts by the tissue paper industry in this area have been directed to optimize each one of these parameters without seriously affecting the others [1]. In the conversion process a commitment is made to establish a balance of these parameters. The final quality required for the product results from a well-balanced combination of its final properties. If a tissue paper product, such as toilet paper, is not smooth enough, increasing the bulk or resistance would not add more value to the product, because softness is the most important property for the end consumer. On the other hand, if this product has an excess of bulk or resistance earlier, there are finishing processes that will improve the softness at the detriment of the loss of one or both properties. As a result, the balance and compromise of the final properties of papers can turn into an improvement in the quality and value for the final product, but generally, some properties are achieved to the detriment of others (see Table 2). With sufficient knowledge of these compensations, the transformation process can be optimized to meet the desired product requirements [2,11].

In this work we intend to study the evolution of mechanical and softness properties of tissue paper varying with the rubber hardness in a laboratory embossing process, using the same industrial base tissue paper. In addition, different combinations of double rubber layers were also studied. A finite element method (FEM) was used to analyze and validate this experiment.

## 2. Materials and Methods

### 2.1. Materials

**Figure 1:**
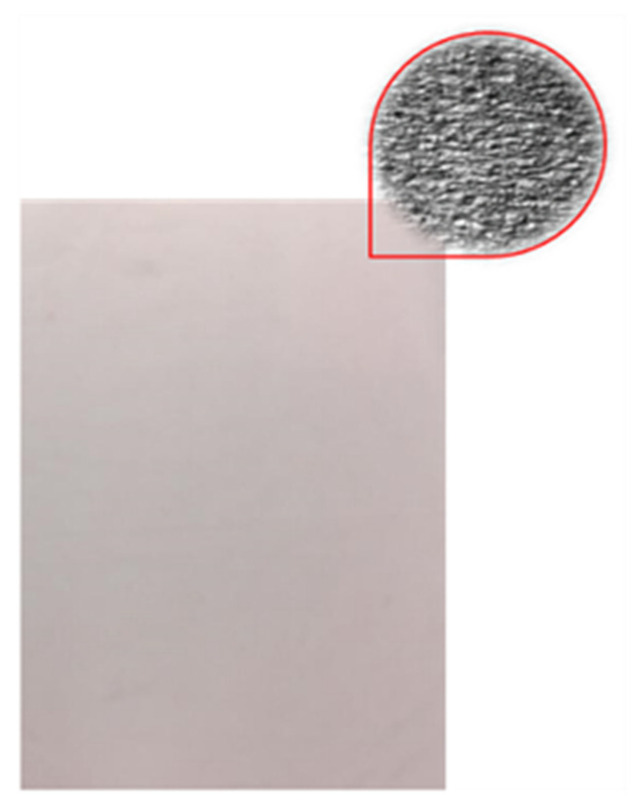
Image of the industrial base tissue paper and the inset showing the crepe structure in a small magnified area.

To perform this work, an industrial base tissue paper (creped paper) from one Portuguese factory was used, as shown in Figure 1. The sample used is composed of a mixture of *eucalyptus globulus* (hardwood) and *pinus* (softwood) bleached kraft pulps, with hardwood content of about 30%, which is fully characterized as sample M in the data article from the authors [12]. This industrial base tissue paper, here designated by sample B, was produced on a machine with single headbox and ceramic creping blade with a grammage of 16.7 g/m^2^.

**Figure 2:**
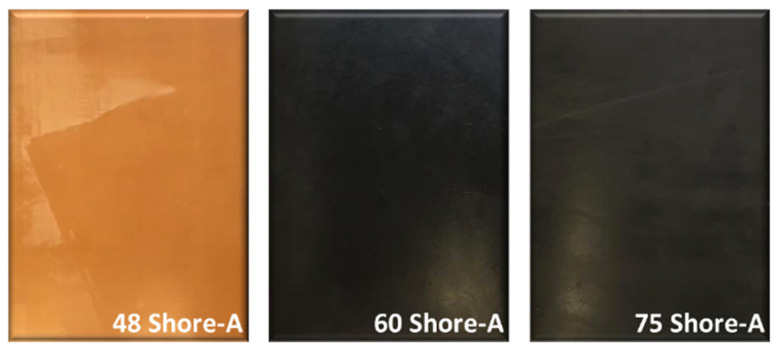
Images of the used rubber plates.

Additionally, for the execution of this work, 3 rubber plates with different hardness and a thickness of 11 mm were chosen, within the range mentioned by the suppliers 50–70 Sh-A. Figure 2 shows the aspect of these rubber plates.

This work began with the determination of rubber hardness according to the specifications provided by the rubber’s supplier. Table 3 presents the hardness results obtained for the rubbers used in this work, as well as other characteristics provided by the supplier.

### 2.2. Methods

This work started by determining the hardness of the three chosen rubbers plates using a PCE-DDA durometer equipment (PCE Iberica S.L. Instrumentacíon, Albacete, Spain), where each rubber plate was divided in 10 sections, and 1 measurement was made in each section, comprising 10 measurements per plate.

**Figure 3:**
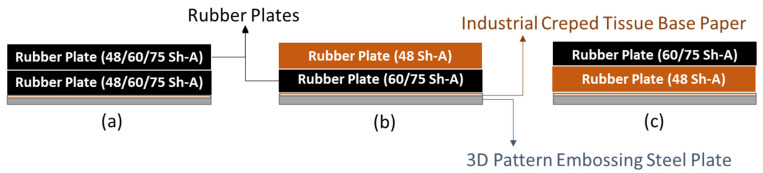
Schematic of the embossing process with different rubber plate hardness configurations: (**a**) configuration 1; (**b**) configuration 2; and (**c**) configuration 3.

Two embossing patterns were used to perform the embossing operation (deco and micro) engraved in steel plates. Furthermore, 3 different configurations of rubber plates were used in this operation, the configurations are exhibited in Figure 3. Configuration 1 consists of a set of the industrial base tissue paper over the embossing steel plate pressured against two rubber plates with same hardness, configuration 2 is the same set pressured against two rubber plates with different hardness (rubber plate with higher hardness in contact with the paper sample), and, finally, configuration 3 is also the same set, but now pressured against two rubber plates with different hardness (rubber plate with lower hardness in contact with the paper sample).

**Figure 4:**
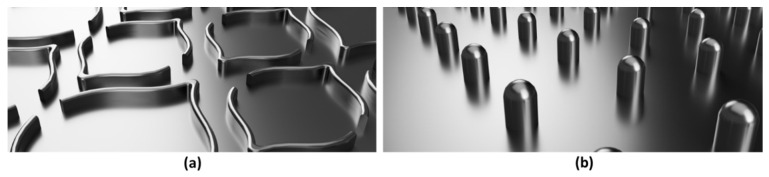
Embossing patterns: (**a**) deco embossing, and (**b**) micro embossing.

The embossing operation conditions used were 2.8 bar during 1 min, for all combinations of 3 stacking configurations and 2 different steel plates, corresponding to the micro and deco embossing patterns. These operating conditions were studied and optimized in a previous work developed by the authors [13]. The two embossing patterns used are shown in Figure 4.

All the samples obtained using both patterns and the 3 rubber staking configurations were tested in terms of thickness/bulk and their mechanical tensile strength in machine direction (MD) and in transverse direction (CD). Tensile tests were performed in a Thwing-Albert**^®^** VantageNX Universal testing machine in accordance with the tissue standard ISO 12625-4:2005 [14]. Thickness and bulk were measured using a FRANK-TPI**^®^** Micrometer accordingly to tissue standard ISO 12625-3:2014 [15]. All samples produced were characterized in terms of softness using the Tissue Softness Analyzer (TSA) from EMTEC**^®^**. The TP II algorithm, and the QA I algorithm were used for the computation of the softness handfeel (HF), respectively.

The conducted experiments for surface image acquisition of paper samples engraved with the embossing patterns in the different considered combinations was carried out using a customized optical system [16,17,18]. This system has shown to be very versatile and useful in this kind of analysis and, therefore, it has already been considered in other related studies [8,9,10,19,20] concerning the visual inspection and quality control of tissue papers.

### 2.3. Finite Element Method (FEM)

A finite element model was proposed to improve the understanding of the effect of rubber stiffness, used in paper manufacturing process, on the mechanical properties. Two different die models were used for simulations (deco and micro) and their effects on the paper stress field and plasticity were analyzed, regarding the rubber stiffness used on the paper embossing process.

To model the paper plasticity, an orthotropic elastic-plastic material model [21] was implemented as a user material subroutine for explicit simulations (VUMAT) which is linked to the commercial finite element software ABAQUS^TM^ version 6.14 (Waltham, MA, USA). This material model allows to account for the paper anisotropic behavior, since paper mechanical behavior is highly dependent of fiber orientation [21]. Since the library of commercial finite element software ABAQUS^TM^ does not have a plasticity constitutive model for anisotropic materials, this VUMAT allows to implement a different constitutive relation based on the isotropic $J_{2}$ model. The model assumes the additive decomposition of the strain tensor into elastic strain tensor plus plastic strain tensor (Equation (1)), considering that volume is conserved:

(1) $$\varepsilon_{ij}=\varepsilon_{ij}^{e}+\varepsilon_{ij}^{p},$$

where $\varepsilon_{ij}$ is the total strain, $\varepsilon_{ij}^{e}$ is the elastic strain, and $\varepsilon_{ij}^{p}$ is the plastic strain.

The model uses the concept of an isotropic plasticity equivalent [22] which is a fictitious material that relates the orthotropic stress state to the isotropic stress state. The relation between the actual Cauchy stress tensor and the isotropic plasticity equivalent (IPE) deviatoric tensor is given in Equation (2):

(2) $$s_{ij}=L_{ijkl}\sigma_{kl},$$

where $s_{ij}$ is the deviatoric IPE stress tensor, $\sigma_{kl}$ is the Cauchy stress and $L_{ijkl}$ is the fourth order transformation tensor shown in Equation (3) for plane stress:

(3) $$L=\left[\begin{array}{ccc} 2A & C-A-B & 0\\ C-A-B & 2B & 0\\ B-C-A & A-B-C & 0\\ 0 & 0 & 3D \end{array}\right],$$

where parameters *A*, *B*, *C,* and *D* are obtained from the experiments using the following equations [21]:

(4) $$A=\sqrt{1-12x^{2}},$$

(5) B=3(y−x),

(6) C=3(y+x),

(7) D=K12n(n+1)3,

(8) $$x=\sqrt{\frac{\alpha^{2}}{24\left(3\alpha^{2}+\beta^{2}-4\beta +4\right)}\left(\beta +1-\sqrt{6\beta -3\alpha^{2}-3}\right)},$$

(9) $$y=\frac{\alpha }{4x}-A,$$

(10) α=K332n(n+1)−K222n(n+1),

(11) β=K332n(n+1)+K222n(n+1),

Parameters $K_{ii}$ and n were obtained by curve fitting the experimental results, applying the Ramberg-Osgood methodology. For MD direction, the constitutive relation that models the tensile test was:

(12) $$\varepsilon_{11}=\frac{\sigma_{11}}{E_{11}}+{\left(\frac{\sigma_{11}}{E_{0}}\right)}^{n},$$

where for the CD direction was:

(13) $$\varepsilon_{kk}=\frac{\sigma_{kk}}{E_{kk}}+{\left(\frac{K_{kk}E_{kk}}{E_{0}}\right)}^{n},k=2,3$$

Note that in Equation (13) the repeated indices do not mean the usual summation rule used in the indicial notation. Finally, the parameter $K_{12}$ is obtained using Equation (14).

(14) $$\gamma_{12}=\frac{\sigma_{12}}{G_{12}}+{\left(\frac{K_{12}\sigma_{12}}{E_{0}}\right)}^{n},$$

The Hooke’s law for plane stress and small strain linear elastic orthotropic material is defined by Equation (15):

(15) $$\sigma =C\varepsilon^{e},$$

where σ is the Cauchy stress tensor, C is the plane stress linear elastic orthotropic constitutive law and $\varepsilon^{e}$ is the small strain elastic tensor.

**Figure 5:**
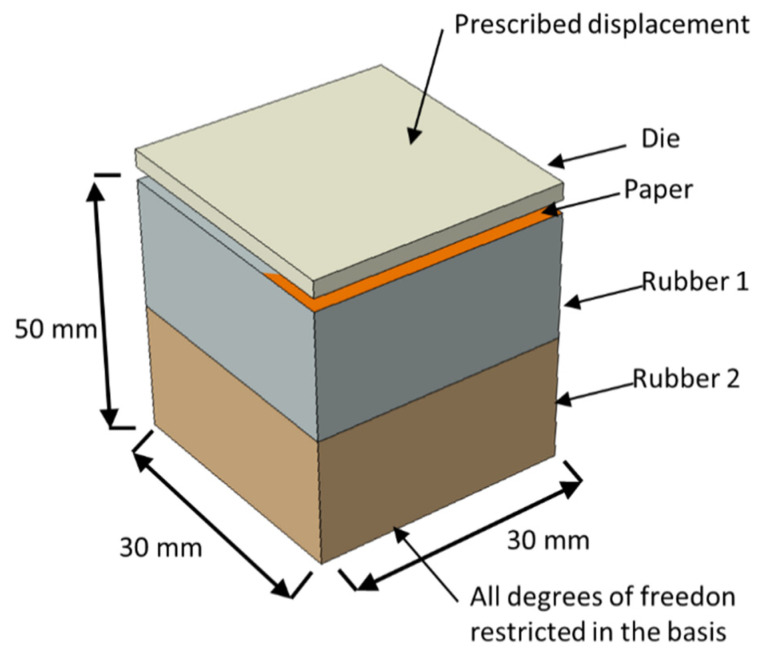
Model dimensions and boundary conditions.

The implementation of this model is similar to the well know $J_{2}$ flow theory for isotropic materials using the backward-Euler algorithm. As mentioned before, this material model was implemented as a user material subroutine for explicit simulations linked to the commercial finite element software ABAQUS^TM^. The explicit solver was used to overcome convergence issues that are usual when using the implicit solver. On the other hand, using the implicit solver, the stable time increment is very small, resulting in long time simulations. The simulations were performed using a workstation with two intel Xeon E5-2630 8 cores (16 cores total with 32 threads) with 256 Gb ram. The finite element model dimensions and boundary conditions are presented in Figure 5. The die model dimensions are representative of the actual die dimensions to result in a reasonable computational cost and keeping the precision capability. The base dimension is the same, but it is thick enough to allow the deformation without severe interference in the die and paper kinematics. The paper follows the same dimensions of the die and the basis.

To simulate the influence of the rubber hardness effect on the paper embossing process, the finite element model of the basis was split into two different parts, allowing to simulate the use of different rubber stacking configuration improving the understanding of the basis stiffness influence on the process.

Regarding the finite element boundary conditions, the bottom of the rubber base was recessed. A prescribed displacement was applied on the top of the die, in the downward direction, while the other degrees of freedom (displacements and rotations) were restricted. For the interactions between the model parts, hard contact for normal behavior and frictionless for tangential behavior was considered. The model has 473,694 elements for the simulation of micro die and 476,153 elements for the deco die. For both models, the paper was simulated using a 4-node reduced integration membrane element (M3D4R with a structured mesh with 250,000 elements. For micro die the 4-node reduced integration element (S4R) was used with total number of elements of 7694 elements. The same element was used for the deco die, but for this die, 10,314 elements were used. Finally, the rubber base was modeled using 8-node tridimensional elements (C3D8) using a total of 216,000 elements.

This model uses 5 different type of materials, steel for the die (E = 200 GPa, μ = 0.33), a hyperelastic isotropic material model for each of the three different types of rubber, CR PR (C10 = 1.8 MPa and D1 = 0.255746 MPa^−1^), SBR PR (C10 = 0.810343 MPa and D1 = 0.56956 MPa^−1^), and NR BG (C10 = 0.409852 MPa and D1 = 1.126109 MPa^−1^), the rubber mechanical properties was estimated relating the hardness with the Young Modulus as suggested by Larsson [23]. Finally, the paper was modeled as an orthotropic material (E11 = 13.89 MPa, E22 = E33 = 4.23 MPa, μ = 0.33 and G12 = 2.1 MPa).

## 3. Results

### 3.1. Configuration 1

**Figure 6:**
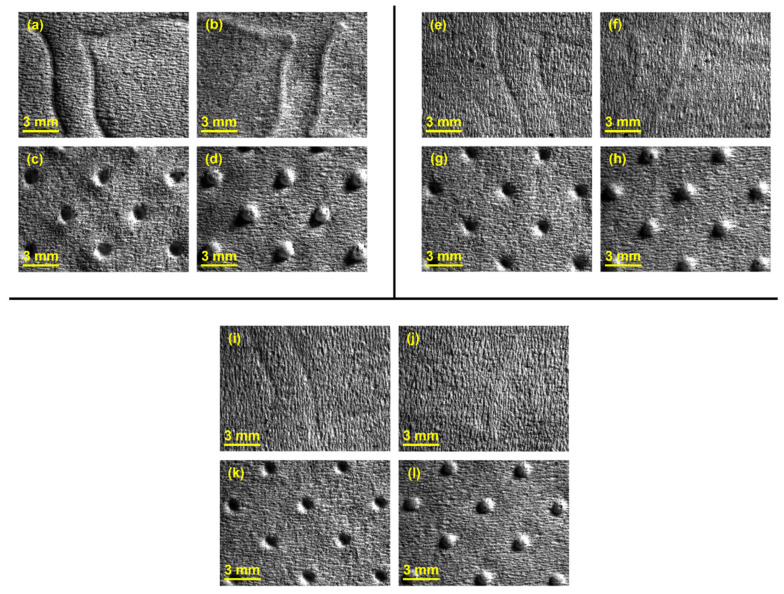
Global views of tissue paper embossed with the different rubber hardness using configuration 1: (**a**) 48 Sh-A (deco—front side); (**b**) 48 Sh-A (deco—back side); (**c**) 48 Sh-A (micro—front side); (**d**) 48 Sh-A (micro—back side); (**e**) 60 Sh-A (deco—front side); (**f**) 60 Sh-A (deco—back side); (**g**) 60 Sh-A (micro—front side); (**h**) 60 Sh-A (micro—back side); (**i**) 75 Sh-A (deco—front side); (**j**) 75 Sh-A (deco—back side); (**k**) 75 Sh-A (micro—front side); and (**l**) 75 Sh-A (micro—back side).

As explained in the previous section, in configuration 1 two rubber plates having the same hardness were stacked. In Figure 6, magnified images of the samples obtained for this configuration can be seen. The papers structure, as well as the engraved patterns performed during the embossing process with different hardness of the rubber plates, can easily be observed.

**Figure 7:**
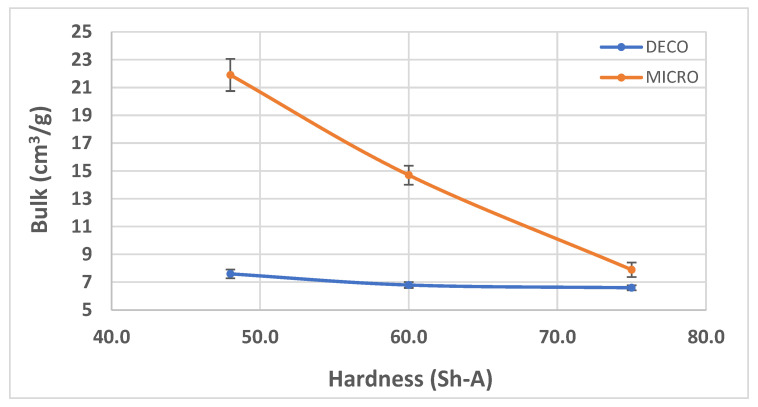
Bulk evolution with the increase in rubber hardness.

**Figure 8:**
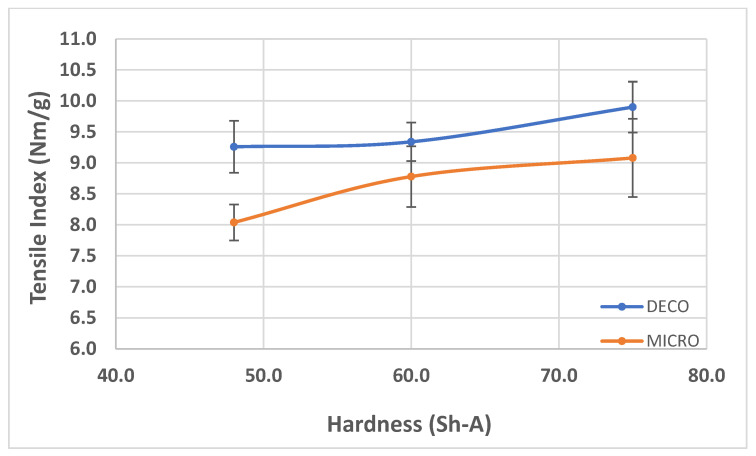
Tensile index behavior with the increase in rubber hardness.

**Figure 9:**
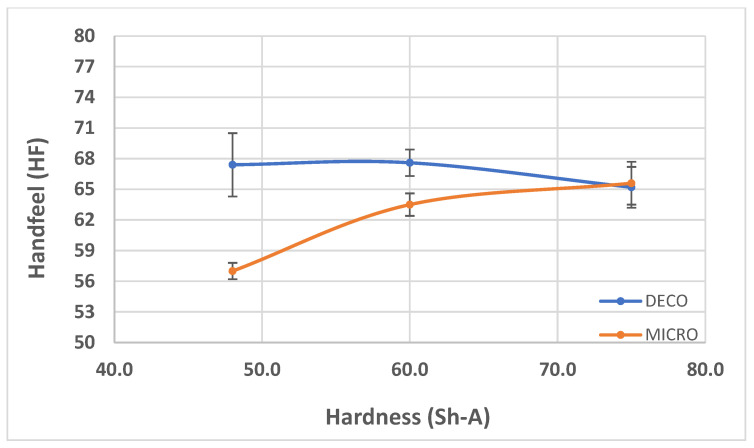
Handfeel comportment with the increase in rubber hardness.

In Figure 7, Figure 8 and Figure 9, the results obtained for the three main properties of tissue paper (bulk, tensile index, and softness, respectively) as a function of rubber hardness for this configuration and both patterns are presented.

### 3.2. Configuration 2

**Figure 10:**
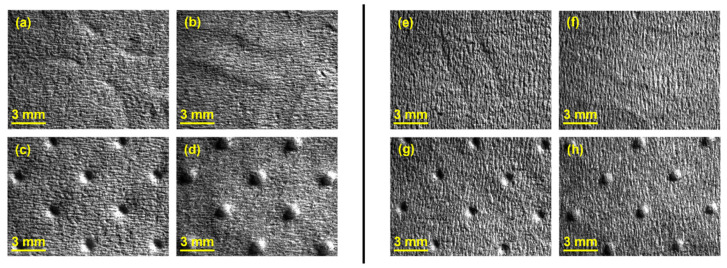
Global views of tissue paper embossed with the different combinations of rubber hardness of configuration 2: (**a**) 60_48 Sh-A (deco—front side); (**b**) 60_48 Sh-A (deco—back side); (**c**) 60_48 Sh-A (micro—front side); (**d**) 60_48 Sh-A (micro—back side); (**e**) 75_48 Sh-A (deco—front side); (**f**) 75_48 Sh-A (deco—back side); (**g**) 75_48 Sh-A (micro—front side); and (**h**) 75_48 Sh-A (micro—back side).

As mentioned before, in configuration 2 two rubber plates with different hardness were stacked, where the rubber plate with higher hardness was in direct contact with the sample. In Figure 10, it can be observed detailed images of the samples obtained for this configuration.

**Figure 11:**
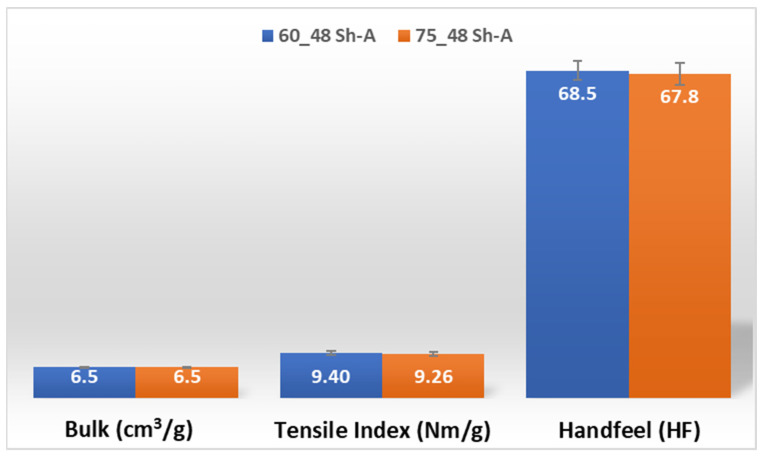
Bulk, tensile index, and handfeel results for deco embossing and configuration 2 rubber hardness conjugation.

**Figure 12:**
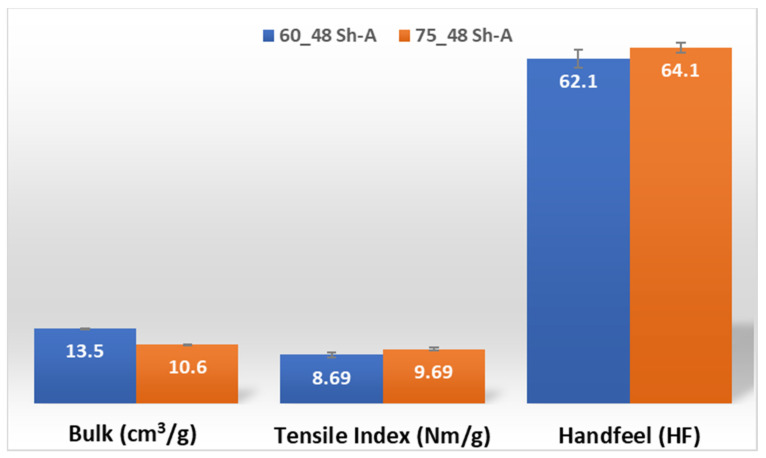
Bulk, tensile index, and handfeel results for micro embossing and configuration 2 rubber hardness conjugation.

In Figure 11 and Figure 12, are shown the results obtained for the three main properties of tissue paper (bulk, tensile index, and softness, respectively) for the two combinations of different rubber hardness plates in configuration 2 and both patterns (deco and micro, respectively).

### 3.3. Configuration 3

**Figure 13:**
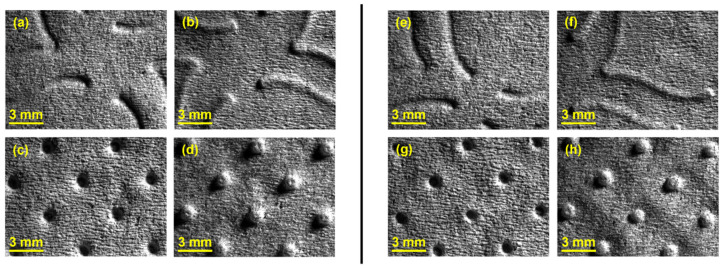
Global views of tissue paper embossed with the different combinations of rubber hardness of configuration 3: (**a**) 48_60 Sh-A (deco—front side); (**b**) 48_60 Sh-A (deco—back side); (**c**) 48_60 Sh-A (micro—front side); (**d**) 48_60 Sh-A (micro—back side); (**e**) 48_75 Sh-A (deco—front side); (**f**) 48_75 Sh-A (deco—back side); (**g**) 48_75 Sh-A (micro—front side); and (**h**) 48_75 Sh-A (micro—back side).

As stated before, in configuration 3 two rubber plates with different hardness were stacked, where the rubber plate with lower hardness was in direct contact with the sample. In Figure 13, detailed images of the samples obtained for this configuration can be seen.

**Figure 14:**
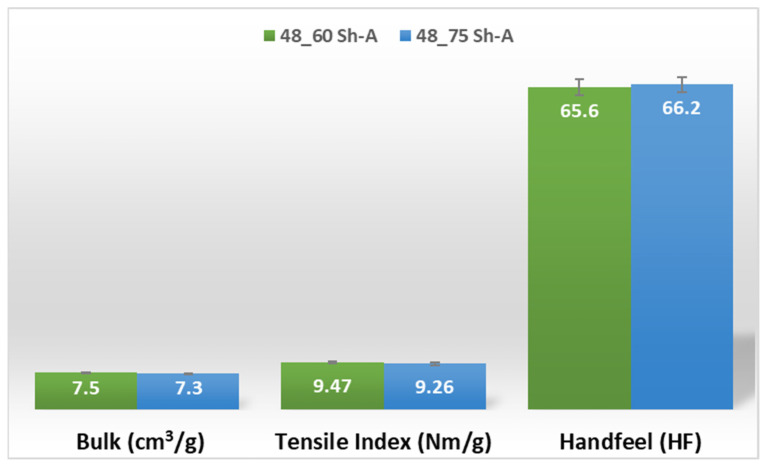
Bulk, tensile index, and handfeel results for deco embossing and configuration 3 rubber hardness conjugation.

**Figure 15:**
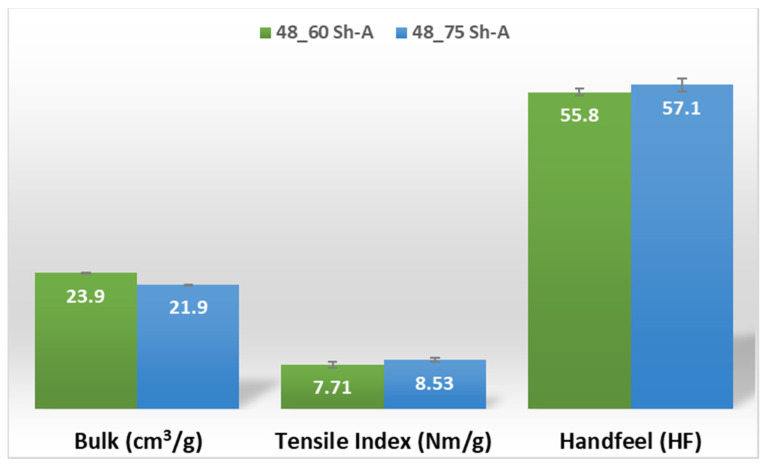
Bulk, tensile index, and handfeel results for micro embossing and configuration 3 rubber hardness conjugation.

The results obtained for the three main properties of tissue paper (bulk, tensile index, and softness, respectively) are shown in Figure 14 and Figure 15, for the two combinations of different rubber hardness plates in configuration 3 and both patterns (deco and micro, respectively).

**Figure 16:**
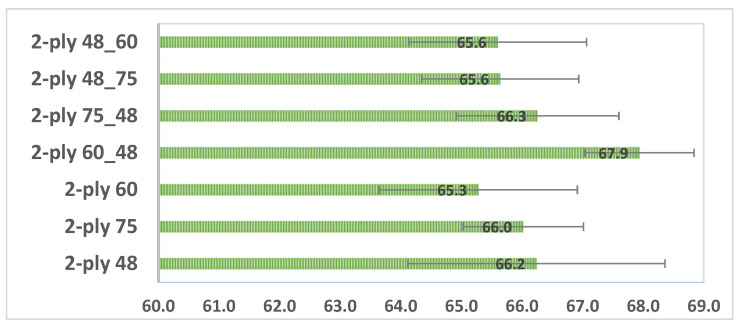
Results obtained for handfeel (HF) with the rubber hardness to the combined 2-ply deco and micro embossed samples.

Combining the deco embossed sheet with the micro embossed sheet for each rubber combination, in each of the three configurations, a prototype of a 2-ply finished product was obtained. With the aim of optimizing the softness as a function of the hardness of the rubber in the embossing process, the handfeel of each one of these prototypes was measured, and the results are shown in Figure 16.

### 3.4. Finite Element Method (FEM)

Several simulations were performed to investigate the effect of the basis rubber hardness regarding the different combinations of rubber and die used for the embossing process on the mechanical properties. This model was calibrated based on experimental results; tensile tests of tissue paper sample B, in both directions (MD and CD) and hardness of three different rubber plates, considering the isotropic Neo-Hookean constitutive model, using three different configurations. However, this model allows, with good precision, to digitally optimize future test with varying configurations and parameters combinations.

#### 3.4.1. Configuration 1

**Figure 17:**
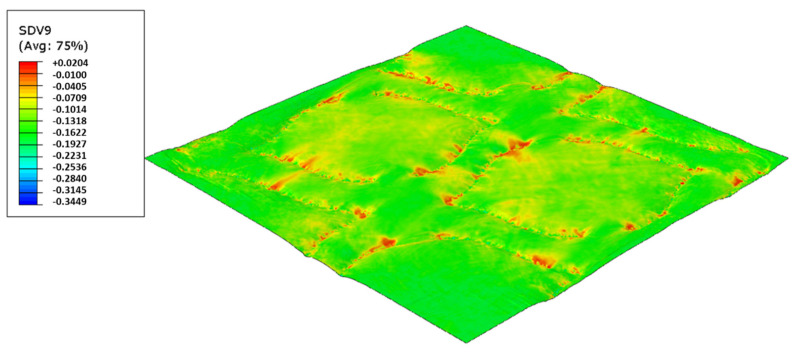
Deco die 75 Sh-A (CR PR), Plastic field.

**Figure 18:**
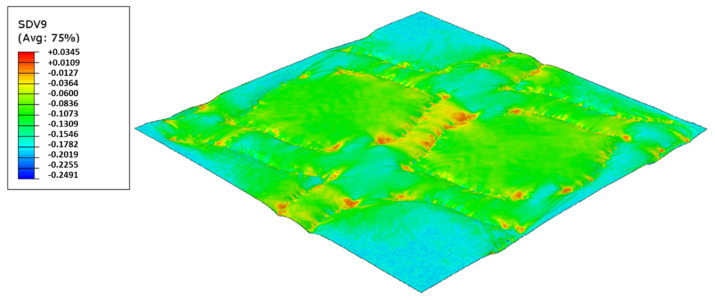
Deco die 60 Sh-A (SBR PR), Plastic field.

**Figure 19:**
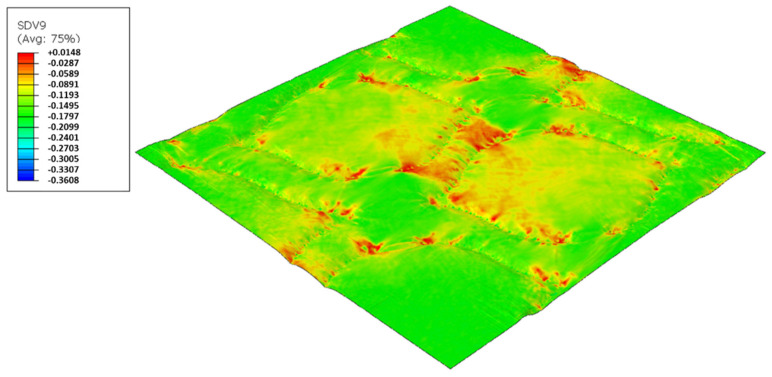
Deco die 48 Sh-A (NR BG), Plastic field.

**Figure 20:**
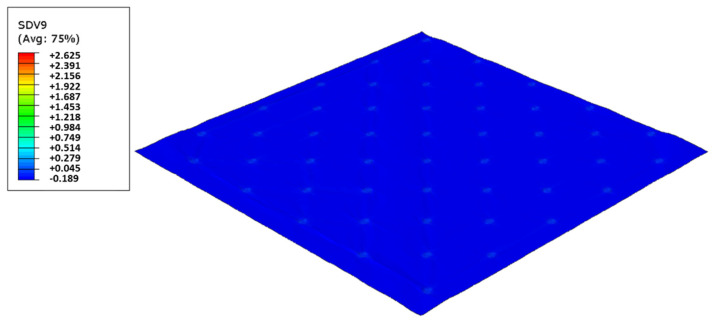
Micro die 75 Sh-A (CR PR), Plastic field.

**Figure 21:**
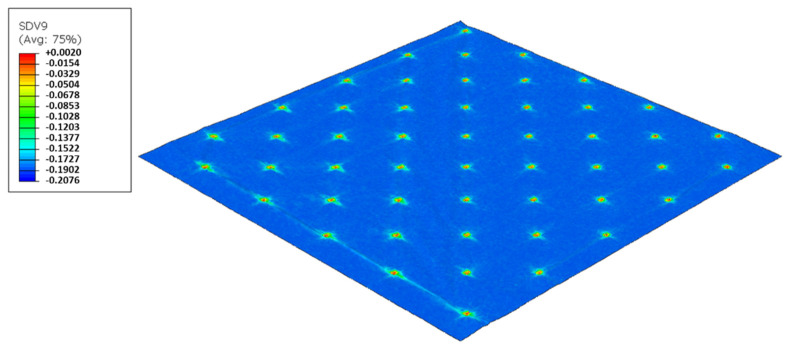
Micro die 60 Sh-A (SBR PR), Plastic field.

**Figure 22:**
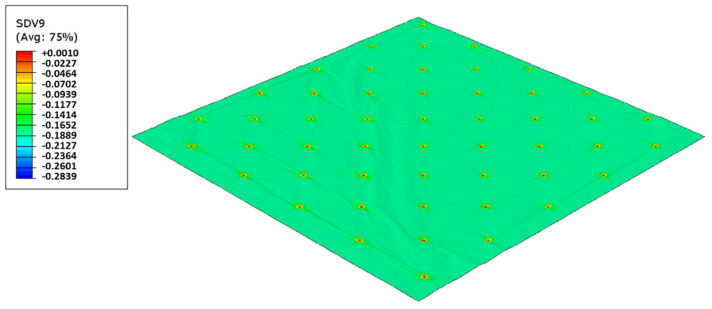
Micro die 48 Sh-A (NR BG), Plastic field.

As used in experiments, configuration 1, uses only one type of rubber in the basis. Thus, three different cases were simulated for the two die models, as showed from Figure 17, Figure 18, Figure 19, Figure 20, Figure 21 and Figure 22.

#### 3.4.2. Configuration 2

**Figure 23:**
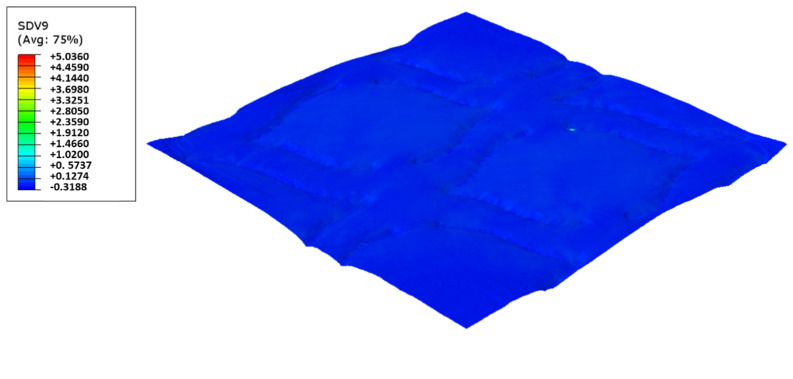
Deco die 75_48 Sh-A (CR PR/NR BG), Plastic field.

**Figure 24:**
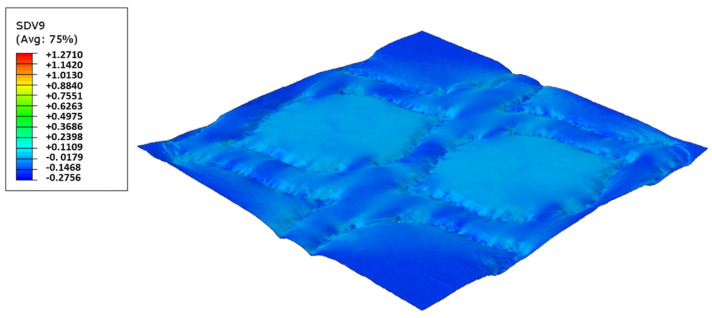
Deco die 60_48 Sh-A (SBR PR/NR BG), Plastic field.

**Figure 25:**
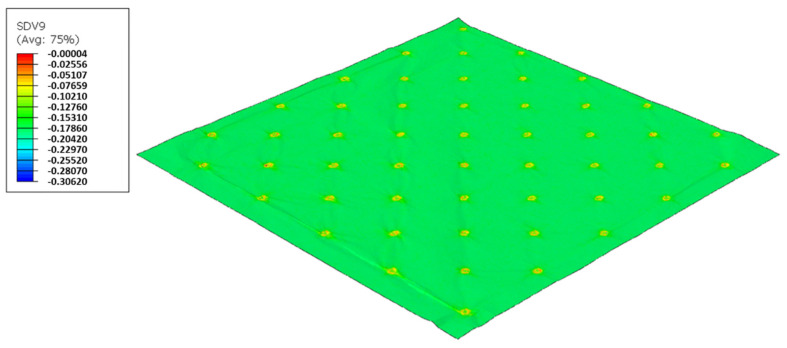
Micro die 75_48 Sh-A (CR PR/NR BG), Plastic field.

**Figure 26:**
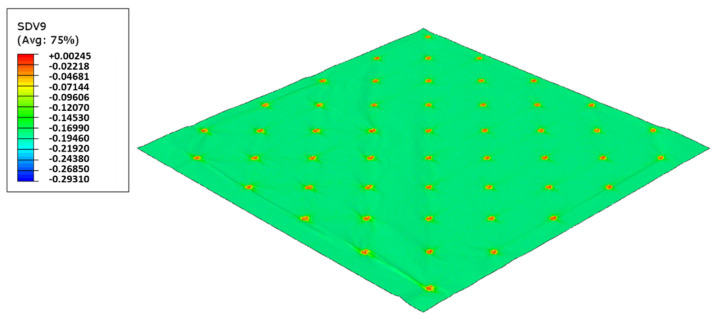
Micro die 60_48 Sh-A (SBR PR/NR BG), Plastic field.

In this configuration, the harder rubber is placed on the top, in direct contact with the paper and the softer rubber is used in the bottom of the elastic basis. Figure 23, Figure 24, Figure 25 and Figure 26 presents the simulation results for the rubber hardness conjugations of configuration 2.

#### 3.4.3. Configuration 3

**Figure 27:**
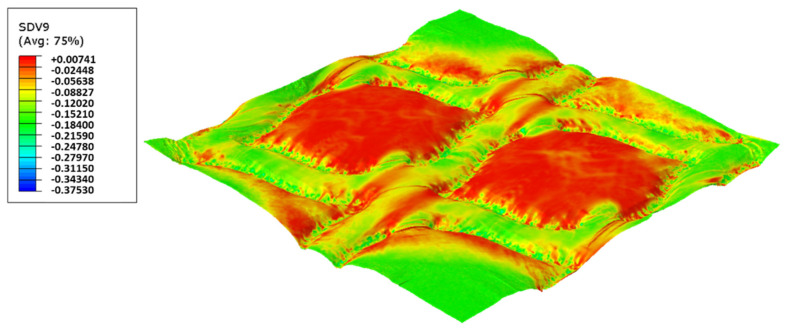
Deco die 48_75 Sh-A (NR BG/CR PR), Plastic field.

**Figure 28:**
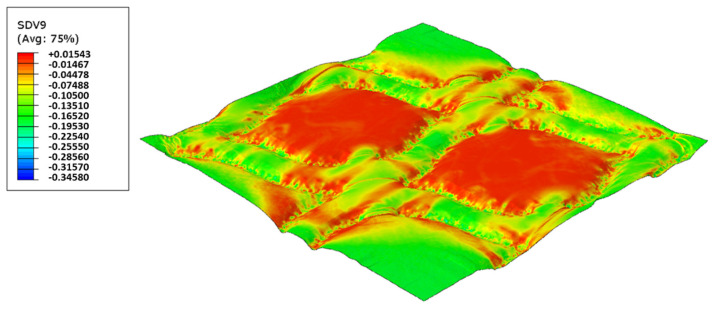
Deco die 48_60 Sh-A (NR BG/SBR PR), Plastic field.

**Figure 29:**
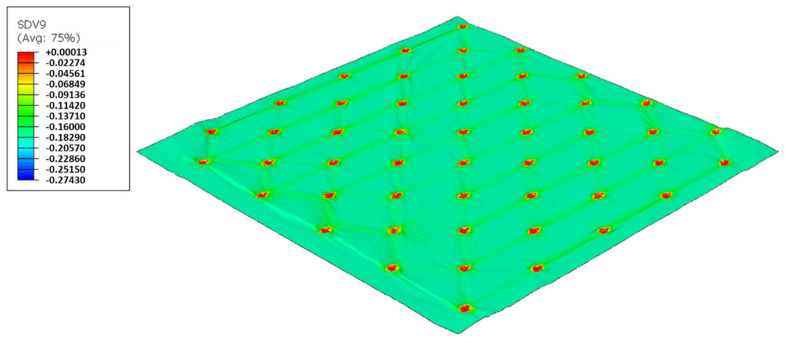
Micro die 48_75 Sh-A (NR BG/CR PR), Plastic field.

**Figure 30:**
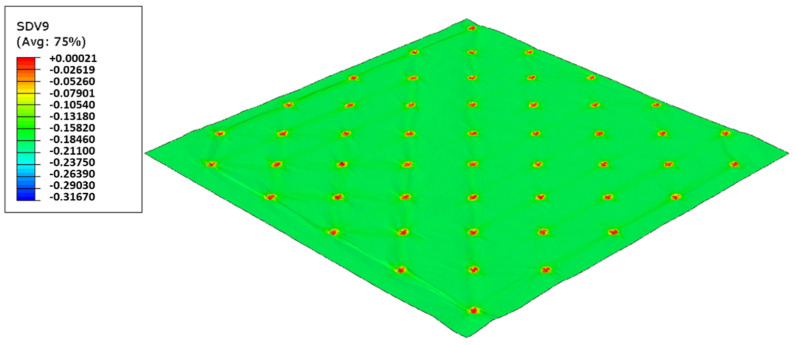
Micro die 48_60 Sh-A (NR BG/SBR PR), Plastic field.

In this configuration, the softer rubber is placed on the top, in direct contact with the paper. Figure 27, Figure 28, Figure 29 and Figure 30 presents the simulation results for the rubber hardness conjugations of configuration 3.

## 4. Discussion

For configuration 1, analyzing the images presented in Figure 6, it can be seen that with the increase in rubber hardness, the marks engraved with the two used embossing patterns appear shallower in the surfaces of the paper. In particular, the marks obtained with the deco embossing are very similar and very dissimulated for the 60 Sh-A and 75 Sh-A hardness’s and the same marks appear a lot more pronounced for the 48 Sh-A hardness. The same effect can also be noticed for the marks obtained with the micro embossing, on which deeper holes are engraved for the 48 Sh-A hardness, and shallower holes are engraved with the 60 Sh-A and 75 Sh-A hardness’s. This effect can also be observed, not only by observation of the formed holes in the images shown in Figure 6c,g,k, but also considering the counter side of the paper surfaces engraved with the micro pattern, shown in the images of Figure 6d,h,l. According to the relations obtained in Figure 6, Figure 7 shows the same tendency for bulk, in which, for both embossing patterns, the bulk decreases with the increase in rubber hardness, and this decrease is more pronounced for the micro pattern. Contrary to bulk, the tensile index grows with the increase in rubber hardness as shown by the graph in Figure 8. From 48 Sh-A to 75 Sh-A rubber hardness, the tensile index has an increase of 6.5% for the deco pattern and 11.5% for the micro pattern. As expected, this increase is more pronounced for the micro pattern, since this pattern, by creating a deeper mark on the tissue paper sheet, it weakens the fibrous structure of the sheet, and, consequently, increases the difference obtained between the rubber hardness extremes. Looking at Figure 9, the handfeel for the deco pattern does not change greatly with increasing hardness of the rubber plates. Contrarily, for the micro pattern, it was verified that with the increase in the rubber plates hardness there is an increase in the handfeel value, since the structure of the paper sheet is less marked for higher hardness of the rubbers. This is in line with the images in Figure 6. Having softness as the main property for the final consumer, and making a compromise between the different properties studied, for configuration 1, the best rubber plate hardness was 60 Sh-A. Figure 17, Figure 18 and Figure 19 show the simulation results for the configuration with 75 Sh-A, 60 Sh-A and 48 Sh-A hardness for the deco pattern. The simulations show that the amount of plasticity found in the paper finite element model is related with the rubber hardness. For the harder rubber (Figure 17), the amount of plasticity (or permanent deformation) is smaller than that found for softer rubber (Figure 18 and Figure 19). For the softer rubber the plastic field is much more visible. Those results corroborate the experiments showed in Figure 6, where the softer rubber allows deeper marks on the paper. Figure 20, Figure 21 and Figure 22 show the simulation results for the configuration with 75 Sh-A, 60 Sh-A, and 48 Sh-A hardness for the micro pattern. As for the deco pattern, the simulations show that the amount of plasticity found in the paper finite element model is related with the rubber hardness. Those results also corroborate the experiments showed in Figure 6, where the softer rubber allows deeper marks on the paper.

For configuration 2, examining the pictures of Figure 10, it can be seen that for the deco embossing, both the considered cases, 60_48 Sh-A and 75_48 Sh-A, reveal marks that show to be engraved in the surfaces of the paper in a similar manner of the marks obtained with the rubber’s hardness of 60 Sh-A and 75 Sh-A alone considered in the configuration 1. In particular, in Figure 6e,f,i,j and Figure 10a,b,e,f, one can observe that the marks reveal to be very shallow on all the mentioned examples. Concerning the micro embossing, the combination of rubbers 60_48 Sh-A, as shown in Figure 10c,d, reveals marks that are very similar with the ones obtained using the rubber 75 Sh-A alone, Figure 6k,l. It is the second combination of rubbers considered in configuration 3, 75_48 Sh-A, that shows the less engraved marks performed with the micro embossing pattern. The great difference of this example comparatively to all the others is remarkable and easily recognizable, as shown in Figure 10g,h. Analyzing the results of Figure 11, for the embossing deco pattern, it can be verified that for bulk, tensile index, and handfeel, the two combinations of rubber plates hardness show very similar values. In Figure 12, for the embossing micro pattern, the results show that for bulk, as expected, the rubber plate in contact with the sheet with less hardness has a higher value, on the other hand, for the tensile index and handfeel, the rubber plate in contact with the sheet with higher hardness originates higher values, since the sheet is less marked by the embossing pattern (see Figure 10) and, consequently, its structure is less affected. As the softness for the end consumer is the key property of the toilet paper, and making a compromise between the different properties studied, for configuration 2 and pattern of embossing deco, the best hardness was 60_48 Sh-A, while for the micro embossing pattern the best hardness was 75_48 Sh-A. In this configuration the plastic field is not detected for the configuration with the harder rubber on top (Figure 23) and a smaller plasticity in configuration with 60 Sh-A rubber on the top (Figure 24). Those results corroborate the experimental results showed in Figure 10 for the deco die. Thus, the basis with softer rubber allows deeper marks on the paper. As for the case of deco die, in the micro die, the plastic field is more pronounced for the case with the softer rubber on top (Figure 26) than for the harder rubber on the top (Figure 25). Those results also corroborate the experimental results showed in Figure 10 for micro die regarding the visual marks on the paper.

Considering configuration 3 and looking at the images presented in Figure 13, it can be seen that both the considered cases, 48_60 Sh-A and 48_75 Sh-A, reveal marks that show to be deeply engraved in the surfaces of the paper, in particular, the first one which results from the combination of the two of the softest rubbers. An interesting analogy can also be made by comparing the images of Figure 13 with the images of Figure 6, on which the marks obtained in the two considered cases of configuration 2 show great similarities with the marks obtained with the use of 48 Sh-A rubber alone in configuration 1. As in configuration 2, the analysis of Figure 14, for the embossing deco pattern, very similar values were found for both bulk and tensile index in the two combinations of rubber plates. Due to the fact that the rubber plate in contact with the sheet has a lower hardness, it will imprint a deeper embossing pattern on the sheet (comparison of the images of Figure 10 with Figure 13) and consequently increases the bulk. Thus, the bulk of configuration 3 is higher than that of configuration 2. Since the operating conditions of the embossing process were optimized for this system in a previous work reported by the authors Vieira et al. 2022 [13], there is a maximization of the mechanical properties by the densification of the sheet structure. Therefore, despite verifying an increase in bulk there is no loss of mechanical strength. Regarding the handfeel, the results obtained in absolute value are lower than those obtained in configuration 2, thus being in accordance with the trend obtained for configuration 1. For the embossing micro pattern, Figure 15 presents a behavior very similar to configuration 2. In absolute value, the bulk was higher, while the tensile index and handfeel were lower. Likewise, this is justified, for the bulk, by the deeper marks of the embossing pattern on the sheet (comparing images of Figure 10 with Figure 13), and for the tensile index, by the densification of the sheet structure which results in the maximization of the mechanical properties. Once the sheet is more marked, there is an increase in the surface roughness decreasing the handfeel value. Since the manufacturer wants the highest softness value with the least loss of other properties, in this configuration of rubber plates, the combination chosen would be 48_75 Sh-A hardness for both embossing patterns. For this case, the simulation showed the results for the softer rubber placed on top of the basis in direct contact with the paper sheet. In this configuration, the plastic field is similar for both cases (Figure 27 and Figure 28). For the case with micro pattern, the plastic field is similar for both cases, but the plasticity detected for the softer rubber configuration (Figure 30) is more pronounced than for the configuration with harder rubber on the bottom of the basis (Figure 29). In any case, when we combine rubbers of different hardness, using the lowest rubber hardness in contact with the paper sheet, this results in an embossed paper with lower softness values. Thus, configuration 3 is not an option for toilet paper production, since softness should be maximized.

From the results shown in Figure 16 it can be inferred that configuration 2 is the best rubber hardness configuration for the embossing process. This result agrees with what is mentioned in the bibliography [3], in which it is stated that the trend for future rubber embossing rollers will have an inner layer with low hardness and an outer layer with higher hardness. This is translated into configuration 2, where the rubber plate in contact with the paper sheet has higher hardness and the other plate has lower hardness. Furthermore, within configuration 2, the prototype 2-ply 60_48 Sh-A is the one that showed the best result for softness.

The finite element simulations results allow understanding the effect of the hardness of the rubber basis (and the combinations of rubbers) on the embossing process and linking the plastic field to the paper marks, where the combination of softer rubber results in deeper marks and, on the opposite, shallow marks (low plasticity) increase the paper strength. The validation of this numerical methodology to virtually optimize the process parameters is a subject for future works. In the present work, with a simple adaptation of the *J*_2_ elastoplastic constitutive model that addresses orthotropy (and numerical implemented in Fortan^TM^ via VUMAT) for the paper, and the isotropic Neo-Hookean constitutive model for the rubbers (available in the library of ABAQUS^TM^) were considered in the FEM model. The calibration of material parameters based on mechanical testing was also addressed. Hence, we can use this numerical methodology for any configuration of rubber laminates with varying thickness and hardness, or other papers based on tensile tests on MD and CD directions.

## 5. Conclusions

This work led us to conclude that configuration 2, is the best solution to obtain a maximum softness. In accordance with this statement the prototype 2-ply 60_48 Sh-A is the one that achieved the highest handfeel.

The obtained results point to an increase in softness with increasing rubber hardness. The mechanical property losses are more pronounced for the lower rubber hardness and for the micro embossing pattern. In contrast, bulk is higher for lower rubber hardness and micro embossing pattern. Consequently, when the hardness of different rubbers is combined and the lower hardness rubber is in contact with the paper sheet, the final properties tend to those of the low hardness rubber alone.

The finite element simulations allow the understanding of how the embossing process and rubber hardness affect the paper characteristics. Thus, finite element models are shown to be a reliable tool to simulate the embossing process for paper industry and predict the final paper mechanical characteristics.

This research has highlighted the importance of the rubber hardness in the embossing process and its impact on the final properties of the tissue paper products.

The authors believe that these results improve the knowledge about the embossing process when the impact of each embossing parameter is studied separately, in this case the hardness of the rubber, and also the quality of the final product. In particular, these findings support the idea pointed out in the introduction that the future trend is the usage of embossing rubber rolls with a low hardness internal layer and a high hardness external layer. In future research other configurations considering the combination of different number of rubber plates will be studied, either by FEM simulation or experimentally.

## Figures and Tables

**Table 1:** Range of hardness and advantages of rubber cover in the embossing process [4,5].

Product	Material	Hardness (Sh-A)	Cover Thickness (mm)	Advantages	Supplier
Skapa Mark Rubber	Rubber Standard	50–70	15–25	Abrasion resistance	Skapa
Skapa Mark Rubber plus	Rubber Premium	50–70	15–25	Better Abrasion resistance	Skapa
Skapa Mark Pur	Polyurethane Standard	50–70	15–25	Good Lifetime	Skapa
Skapa Mark Ultra	Polyurethane Premium	50–70	15–25	Optimized Lifetime; Marking Intensity	Skapa
EmboFlex Plus	Rubber Premium	60	12–20	Good resistance	Hannecard
EmboFlex XL	Rubber Premium	50–70	12–20	Good resistance	Hannecard
Multiplast	Rubber Premium	55	10–20	Demanding Applications	Hannecard
Resistoplast	Rubber Premium	45–98	10–20	Demanding Applications	Hannecard
Resistoplast-AS	Rubber Premium	55	10–20	Antistatic	Hannecard
XL Plast	Rubber Premium	50–95	10–20	Demanding Applications	Hannecard

**Table 2:** Main effects of finishing processes on paper properties [11].

Type of Finishing Process	Scheme of Finishing Process	Gain Properties	Loss Properties
Calendering	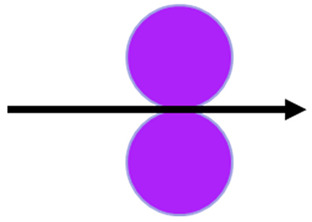	Softness	Bulk
Embossing	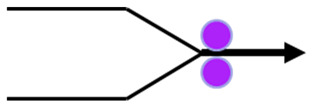	Bulk; Absorption Capacity; Softness	Tensile Strength; Softness
Ply Bonding and Laminating	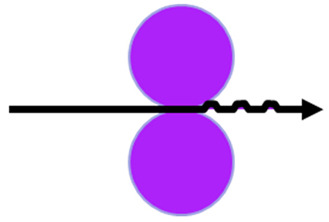	Absorption Capacity; Softness; Bulk (in some cases)	Softness
Lotionizing	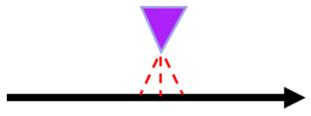	Softness	Absorption Capacity
Printing	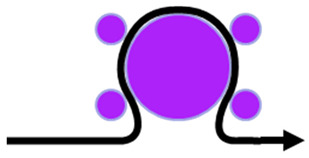	Aesthetic Appearance	Bulk

**Table 3:** Rubber plates characterization results.

Rubber Characteristics	NR BG (45 ± 5 Sh-A)	SBR PR (65 ± 5 Sh-A)	CR PR (70 ± 5 Sh-A)
Hardness (Sh-A)	47.7 ± 0.55	60.3 ± 1.21	75.1 ± 1.98
Tensile Strength (MPa)	16.0	3.0	4.0
Density (g/cm^3^)	1.05	1.6	1.5
Operation Temperature (°C)	−40 to +85	−20 to +100	−20 to +100

## Data Availability

Not applicable.
